# Fabrication of Flexible Arrayed Lactate Biosensor Based on Immobilizing LDH-NAD^+^ on NiO Film Modified by GO and MBs

**DOI:** 10.3390/s17071618

**Published:** 2017-07-12

**Authors:** Jung-Chuan Chou, Siao-Jie Yan, Yi-Hung Liao, Chih-Hsien Lai, You-Xiang Wu, Cian-Yi Wu, Hsiang-Yi Chen, Hong-Yu Huang, Tong-Yu Wu

**Affiliations:** 1 Graduate School of Electronic Engineering, National Yunlin University of Science and Technology, Douliu 64002, Taiwan; M10313322@yuntech.edu.tw (S.-J.Y.); chlai@yuntech.edu.tw (C.-H.L.); M10513317@yuntech.edu.tw (Y.-X.W.); M10513328@yuntech.edu.tw (C.-Y.W.); 2 Department of Electronic Engineering, National Yunlin University of Science and Technology, Douliu 64002, Taiwan; B10213110@yuntech.edu.tw (H.-Y.C.); B10200017@yuntech.edu.tw (H.-Y.H.); 3 Department of Information and Electronic Commerce Management, TransWorld University, Douliu 64063, Taiwan; liaoih@twu.edu.tw; 4 Graduate School of Mechanical Engineering, National Yunlin University of Science and Technology, Douliu 64002, Taiwan; D10011005@yuntech.edu.tw

**Keywords:** lactate biosensor, nickel oxide (NiO), graphene oxide (GO), magnetic beads (MBs), electrochemical impedance spectroscopy (EIS)

## Abstract

We proposed the flexible arrayed lactate biosensor based on immobilizing l-lactate dehydrogenase (LDH) and nicotinamide adenine dinucleotide (${\mathrm{NAD}}^{+}$) on nickel oxide (NiO) film, and which the average sensitivity could be enhanced by using graphene oxide (GO) and magnetic beads (MBs). By using GO and MBs, it exhibits excellent sensitivity (45.397 mV/mM) with a linearity of 0.992 in a range of 0.2 mM to 3 mM. According to the results of electrochemical impedance spectroscopy (EIS), the electron transfer resistance of LDH-${\mathrm{NAD}}^{+}$-MBs/GPTS/GO/NiO film was smaller than those of LDH-NAD^+^/GPTS/GO/NiO film and LDH-${\mathrm{NAD}}^{+}$/GPTS/NiO film, and it presented the outstanding electron transfer ability. After that, the limit of detection, anti-interference effect and bending test were also investigated.

## 1. Introduction

Beyond glucose, blood lactate monitoring is also greatly important in critical care medicine [1]. In several biochemical processes that involved muscle movement, lactate plays an important role, and it is the key metabolite in the anaerobic glycolytic pathway [2,3]. When the energy in tissues is insufficient from aerobic respiration, an increase in lactate concentration will occur from the anaerobic metabolism [4]. The normal range of lactate concentration in human blood is from 0.5 mM to 1.5 mM [2]. The lactate monitoring technology has been developed and used in terms of clinical diagnostics, sports medicine, and food analysis [1,2,3,4,5].

l-lactate dehydrogenase (LDH) is an enzyme, which presents throughout the tissues such as blood cells and heart muscle, and it has a high catalytic activity for conversion of lactate with an aid of a coenzyme (nicotinamide adenine dinucleotide, ${\mathrm{NAD}}^{+}$). Recently, LDH and ${\mathrm{NAD}}^{+}$ have widely been used to catalyze reaction of lactate, and which are mainly on the purpose of lactate monitoring. Moreover, the catalytic reaction mechanism of LDH could be described by the following equations [2]:

(1) $${\mathrm{Lactate}\;+\;\mathrm{NAD}}^{+}\;\overset{\;\mathrm{LDH}\;}{\to }{\;\mathrm{Pyruvate}\;+\;\mathrm{NADH}\;+\;H}^{+}$$

(2) $$\mathrm{NADH}\;\overset{\;}{\to }{\;\mathrm{NAD}}^{+}{\;+\;H}^{+}{\;+\;2e}^{-}$$

where NADH is reduced form of ${\mathrm{NAD}}^{+}$, $H^{+}$ is positively charged hydrogen ion and $e^{-}$ is electron. According to the above equations, LDH catalyzes the conversion of lactate and ${\mathrm{NAD}}^{+}$ to pyruvate, NADH and $H^{+}$. Simultaneously, NADH is oxidized into ${\mathrm{NAD}}^{+}$, $H^{+}$ and ${2e}^{-}$ when external energy is obtained.

Nickel oxide (NiO) is one of the p-type semiconducting materials. NiO has been used in biomonitoring such as glucose, uric acid, urea and lactate monitoring, because it possesses several outstanding advantages such as high chemical stability, biocompatibility, high electron transfer feature, nontoxicity and high electrocatalytic properties [6,7,8,9]. In addition, graphene oxide (GO) and magnetic beads (MBs) are very promising for opening new possibilities in the development of biosensors. It is attributed to not only high specific surface area, good water dispersibility, biocompatibility, high electron transfer ability and high affinity for specific biomolecules of GO [10,11,12], but also biocompatibility, high specific surface area, less toxicity, physicochemical stability, easy functionalization, magneto-controlled location and transport, and an excellent contact between biocatalyst and substrate of MBs [13,14,15].

Lactate is a main indicator for intensity of exercise. Because lactate is generated in the body during exercise, it is widely used for monitoring the level of exercise. Therefore, a wearable sensor is a new tendency in monitoring health status, exercise, etc. [16]. In this study, we used screen-printing technique to fabricate the array of conductive wires and a pair of differential reference electrodes, and integrated them in the lactate biosensor based on flexible plastic substrate. The screen-printed silver reference electrode could replace traditional glass reference electrode, and it also offered advantages such as simplicity of use, small ohmic resistance effect, less contamination, and no liquid junction potential [17]. Moreover, the sensing film of lactate biosensor, proposed by us in this study, was based on NiO film, on which LDH and ${\mathrm{NAD}}^{+}$ were co-immobilized by 3-Glycidoxypropyltrimethoxysilane (GPTS). To enhance the average sensitivity of the lactate biosensor, GO and MBs were used to modify the lactate biosensor. Except it was investigated how much the contents of GO and MBs affected average sensitivity of such lactate biosensor, and the electrochemical impedances of films were analyzed by using electrochemical impedance spectroscopy (EIS), such as LDH-${\mathrm{NAD}}^{+}$/GPTS/NiO film, LDH-${\mathrm{NAD}}^{+}$/GPTS/GO/NiO film and LDH-${\mathrm{NAD}}^{+}$-MBs/GPTS/GO/NiO film. After that, the limit of detection, anti-interference effect and bending test were also investigated.

## 2. Experimental Details

### 2.1. Materials and Reagents

The polyethylene terephthalate (PET) substrate was purchased from Zencatec Corporation (Taoyuan, Taiwan). Epoxy (product No. JA643), composed of epoxy resin, prepolymer, epoxy hardener, accelerator and fumed silica, was purchased from Sil-More Industrial Ltd. (Taipei, Taiwan). Silver paste was purchased from Advanced Electronic Material Inc. (Tainan, Taiwan). Nickel oxide (NiO) target with 99.95% purity was purchased from Ultimate Materials Technology Co., Ltd. (Zhubei, Taiwan). Phosphate monobasic (${\mathrm{KH}}_{2}{\mathrm{PO}}_{4}$) powders and potassium phosphate dibasic ($K_{2}{\mathrm{HPO}}_{4}$) powders were purchased from Darmstadt, Germany and Katayama Chemical Co., Ltd. (Osaka, Japan), were used to prepare 20 mM phosphate buffer solution (PBS) with pH 7. Lactate, L-lactate dehydrogenase (LDH) and β-Nicotinamide adenine dinucleotide hydrate $({\mathrm{NAD}}^{+}),$ 3-Glycidoxypropyltrimethoxysilane (GPTS), toluene and *N*-Ethyl-*N*′-(3-dimethylaminopropyl) carbodiimide hydrochloride (EDC) were purchased from Sigma-Aldrich Co. (Saint Louis, MO, USA). Graphene oxide (GO) powders were obtained from the team of Y.H. Nien [18]. Magnetic bead (MB) solution was purchased from Quantum Biotechnology Inc. (Taichung, Taiwan).

### 2.2. Fabrication of Flexible Arrayed Lactate Biosensor

**Figure 1:**
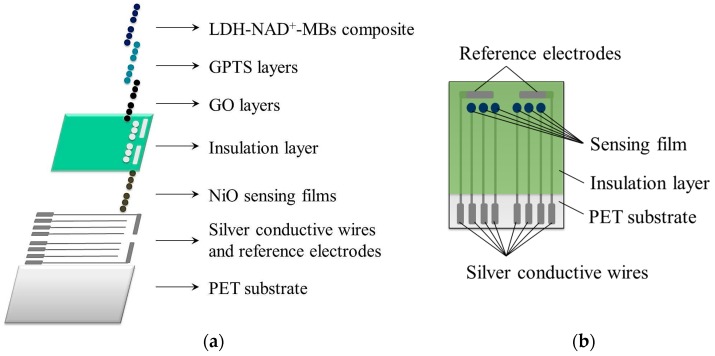
(**a**) The fabrication process and (**b**) the schematic diagram of flexible arrayed lactate biosensor.

**Figure 2:**
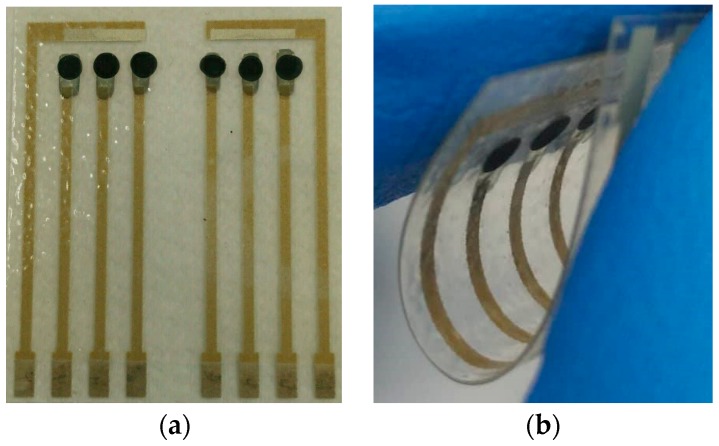
The photos of flexible arrayed lactate biosensor under (**a**) unfolded state and (**b**) bended state.

The fabrication process for flexible arrayed lactate biosensor was shown in Figure 1a, and the detailed descriptions were as follows:

1. The silver paste, used as the conductive wires and reference electrodes, was printed on flexible PET substrate (30 mm × 40 mm) by using screen-printing technique, and was then baked at 120 °C for 30 min in the oven. The screen-printing technique could make traditional glass reference electrode replaced by silver reference electrodes, and it also could achieve the miniaturization of the device.
2. NiO films were deposited on the ends of silver conductive wires by using radio frequency (R.F.) sputtering system. The flow rates of Ar and $O_{2}$ gases were controlled at 10 sccm and 3.8 sccm, respectively. The sputtering power and working pressure were set at 50 watts and 3 mTorr, respectively. The deposition time was 50 min. Furthermore, a pre-sputtering of target in pure Ar atmosphere was done for 10 min to avoid target contamination.
3. Epoxy was printed on part of silver conductive wires by using screen-printing technique, and was then baked at 120 °C for 90 min in the oven. Epoxy was used as an insulation layer to prevent silver conductive wires from corroding from test solution; besides, it was also used to define the areas of sensing windows (1.77 mm^2^). The uncovered silver conductive wires could be connected to potentiometric measurement system, which offered a favor to measurement signal transmission. The potential signals of six sensing windows and two reference electrodes could be obtained by potentiometric measurement system.
4. GO powders were added into deionized (D.I.) water to prepare 0.1 wt %, 0.3 wt % and 0.5 wt % GO solutions, and which were uniformly dispersed by using an ultrasonic vibrator. After that, each 2 μL GO solution was dropped onto NiO film of flexible arrayed pH sensor, and it was left at room temperature for one day.
5. Each 2 μL GPTS-toluene mixture (the volume ratio set at 1:2) was dropped on GO layer, and then it was left in the oven at 120 °C for one hour.
6. Different amounts of MB solutions (10 mg/mL), such as 0.25 mL, 0.50 mL, 0.75 mL and 1.00 mL, were used to investigate the impact of MB contents on average sensitivities of the biosensors.
  - MB solution was drawn out and put into test tube by using micropipettor, and then the suspension in MB solution was drawn out with the aid of external magnetic field.
  - MBs were thrice cleaned with PBS.
  - MBs and EDC solution (10 mg/mL in PBS solution) were mixed and ultrasonicated for 30 min. EDC was used as a carboxyl activating agent to bind MB and enzyme.
  - EDC solution in mixture was drawn out with the aid of external magnetic field so that MBs were obtained.
7. 1 mL enzyme solution, composed of 1 mg LDH, 1 mg ${\mathrm{NAD}}^{+}$ and 100 μL PBS, was added into test tube containing MBs. LDH-${\mathrm{NAD}}^{+}$-MBs composite solution was stirred by the stirrer at 4 °C for 8 h. Each 2 μL LDH-${\mathrm{NAD}}^{+}$-MBs composite solution was dropped on GPTS layer, and was then stored in refrigerator at 4 °C for one day.
8. After that, the flexible arrayed lactate biosensor was completed, the schematic diagram of which was shown in Figure 1b. Moreover, Figure 2a,b showed the photos of flexible arrayed lactate biosensor under unfolded state and bended state.

### 2.3. Measurement

Except measuring the limit of detection, all of the measurements were operated between 0.2 mM and 3 mM lactate solutions because it was the best linear region and also fell within the normal range of lactate concentration in human blood. Therefore, the lactate biosensors were respectively immersed into 0.2, 0.7, 1.3, 2 and 3 mM lactate solutions, and then their response voltages were measured by using potentiometric measurement system. The potentiometric measurement system consisted of the instrumentation amplifiers (LT1167), a data acquisition (DAQ) card and a computer equipped with LabVIEW software. All of the measurement environments were kept at room temperature. All of the average sensitivity and linearity were calculated by calculation program of Origin 7.0.

The electrochemical impedances were analyzed by using EIS. The electrochemical instrument consisted of a potentiostat (SP-150, Bio-Logic Science Instruments, Seyssinet-Pariset, France), a computer with EC-Lab^®^ software and three-electrode setup. The three-electrode setup is made up of a working electrode (sensing film), a platinum (Pt) counter electrode and a commercial silver/silver chloride reference electrode, and the above electrodes are all contacted with electrolyte. A potentiostat with the aid of a computer can supply a stable AC sinusoidal signal by a commercial silver/silver chloride reference electrode, resulting in an electric field that can be generated between working electrode and counter electrode. All of the experiments for EIS used a small AC signal at frequency ranging from 10 kHz to 100 mHz. Moreover, the area and distance of film and Pt counter electrode were kept constant, and all of the measurement environments were kept at room temperature.

In potentiometric measurement system, the potential signals of silver reference electrodes were sent to a computer equipped with LabVIEW through LT1167 and DAQ. Therefore, we could confirm the stability of reference potential. However, in EIS measurement, in order to confirm the stability of the electrochemical reaction, we used a commercial silver/silver chloride reference electrode instead of our silver reference electrodes.

## 3. Results and Discussion

### 3.1. Characterization of NiO Film

**Figure 3:**
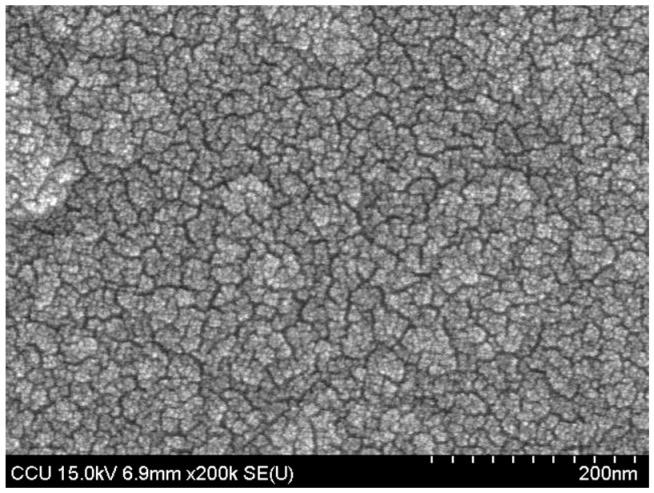
The surface morphology of NiO film.

The surface morphology of NiO sensing film was characterized by using a field emission scanning electron microscope (FE-SEM, S4800-I, Hitachi, Tokyo, Japan) equipped with energy dispersive X-ray spectroscopy (EDX) detector with X-ray mapping capability in National Chung Cheng University, Taiwan. Figure 3 showed the surface morphology of NiO film, and it was dense and porous. Besides, from SEM and EDX results, we could know some information about NiO film. The thickness of NiO film was about 355 nm, and the elemental ratio of Ni/O was about 38.50/61.50 = 0.63.

Besides, the performances of NiO film, including mobility, sheet hole concentration and resistivity, were also measured by Hall effect measurement system (HMS-3000), which came from the Precision Instrument Development Center, National Yunlin University of Science and Technology, Taiwan. The measurement parameters were set at a current of 1 mA, the thickness of 355 nm and a magnetic flux density (B) of 0.55 T. The mobility, hole concentration and resistivity of NiO thin film were 1.275 × 10^1^ cm^2^/V∙S, 1.782 × 10^19^ cm^−3^ and 2.748 × 10^−^^2^ Ω·cm, respectively.

### 3.2. Optimization of Flexible Arrayed Lactate Biosensor by Varying GO Content

In this study, GO was used to modify the surface of NiO film because of its high specific surface area, high electron transfer ability and high affinity for specific biomolecules [10,11,12]. Moreover, 0.1, 0.3 and 0.5 wt % GO solutions were used for the processing of film surface modification.

**Figure 4:**
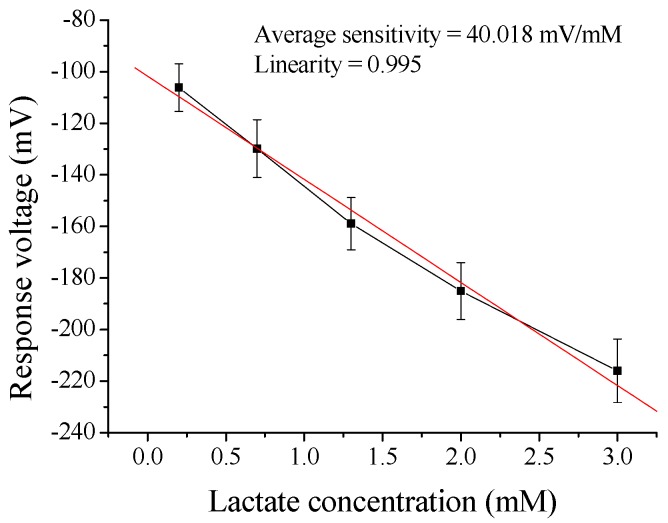
The response voltages of flexible arrayed lactate biosensor modified by 0.3 wt % GO.

The response voltage was measured by potentiometric measurement system. The average sensitivity and linearity of flexible arrayed lactate biosensor based on LDH-${\mathrm{NAD}}^{+}$/GPTS/NiO film were 38.218 mV/mM and 0.992, respectively, as shown in Table 1. Moreover, the experimental results of lactate biosensors based on LDH-${\mathrm{NAD}}^{+}$/GPTS/GO/NiO film were shown in Table 1. The average sensitivity and linearity of flexible arrayed lactate biosensor modified by 0.1 wt % GO were 39.237 mV/mM and 0.998, respectively. The average sensitivity and linearity of flexible arrayed lactate biosensor modified by 0.3 wt % GO were 40.018 mV/mM and 0.995, respectively. However, the average sensitivity and linearity of flexible arrayed lactate biosensor modified by 0.5 wt % GO were 37.146 mV/mM and 0.994, respectively. From Table 1, we could know that the optimal GO content was 0.3 wt % for flexible arrayed lactate biosensor, and which response voltages to different lactate concentrations were shown in Figure 4.

### 3.3. Optimization of Flexible Arrayed Lactate Biosensor by Varying MB Content

After finding the optimal GO content, MBs were used to enhance enzyme-immobilization ability and catalytic ability because of their biocompatibility, high specific surface area, less toxicity, physicochemical stability, easy functionalization, magneto-controlled location and transport, and an excellent contact between biocatalyst and substrate [13,14,15]. Moreover, 0.25, 0.50, 0.75 and 1 mL MBs were respectively mixed into enzyme solution to deposit on the optimal GO layer.

**Figure 5:**
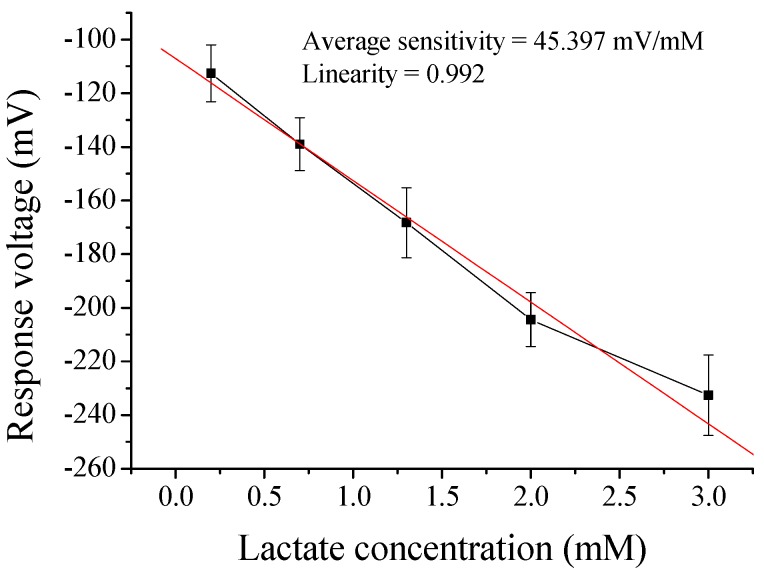
The response voltages of flexible arrayed lactate biosensor modified by 0.75 mL MBs.

The response voltage was measured by potentiometric measurement system. The average sensitivity and linearity of flexible arrayed lactate biosensor based on LDH-${\mathrm{NAD}}^{+}$/GPTS/GO/NiO film were 40.018 mV/mM and 0.995, respectively, as shown in Table 1. On the other hand, the experimental results of lactate biosensors based on LDH-${\mathrm{NAD}}^{+}$-MBs/GPTS/GO/NiO film were shown in Table 2. The average sensitivity and linearity of flexible arrayed lactate biosensor modified by 0.25 mL MBs were 42.531 mV/mM and 0.987, respectively. The average sensitivity and linearity of flexible arrayed lactate biosensor modified by 0.50 mL MBs were 43.595 mV/mM and 0.993, respectively. The average sensitivity and linearity of flexible arrayed lactate biosensor modified by 0.75 mL MBs were 45.397 mV/mM and 0.992, respectively. However, the average sensitivity and linearity of flexible arrayed lactate biosensor modified by 1.00 mL MBs were 43.606 mV/mM and 0.992, respectively. The average sensitivity was increased with increasing MB content but the increase had a limit. The average sensitivity was decreased when MB content was over 0.75 mL. From Table 2, we could know the optimal MB content was 0.75 mL, and which could achieve the highest average sensitivity (45.397 mV/mM), as shown in Figure 5.

### 3.4. Analysis of Electrochemical Impedances for Different Films

**Figure 6:**
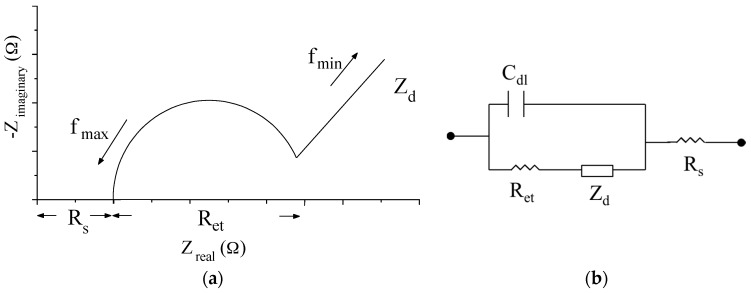
(**a**) The Nyquist diagram and (**b**) its equivalent circuit for biosensor.

In this study, the electrochemical impedances of modified and unmodified films were analyzed to investigate their electron transfer abilities. The electrochemical impedance spectroscopy (EIS) is broadly employed to the descriptions of fundamental electrochemical and electronic processes [19]. The Nyquist diagram and its equivalent circuit for electrochemical biosensor are shown in Figure 6a,b. $R_{\mathrm{et}}$ and $R_{s}$ are the electron transfer resistance and solution resistance, respectively. $C_{\mathrm{dl}}$ is the double layer capacitance. $Z_{d}$ is the diffusion impedance. Figure 6a showed a curve that consists of many complex impedances as function of frequency. In Cartesian coordinates, the real part and imaginary part are plotted on the X and Y axes, respectively. The left semicircle in high frequency is attributed to the electron transfer process, and its diameter is $R_{\mathrm{et}}$. The right arc in low frequency is attributed to the diffusion process.

**Figure 7:**
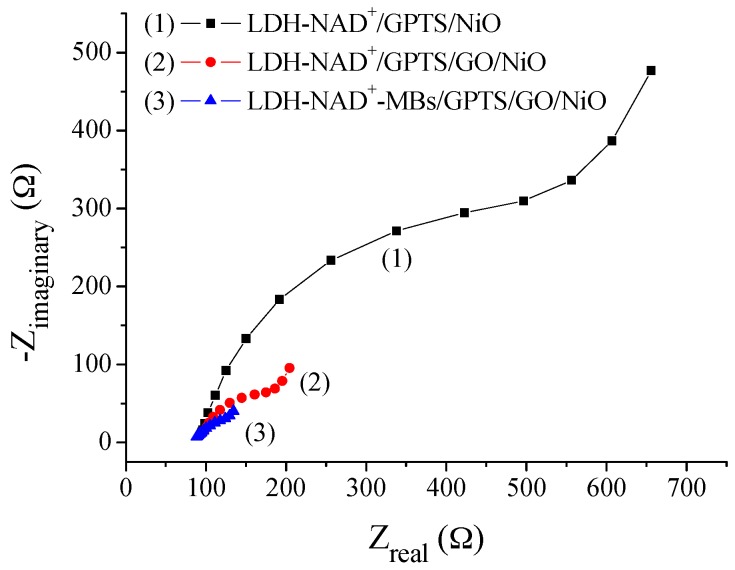
The Nyquist diagrams of flexible arrayed lactate biosensors based on (1) LDH-${\mathrm{NAD}}^{+}$/GPTS/NiO, (2) LDH-${\mathrm{NAD}}^{+}$/GPTS/GO/NiO and (3) LDH-${\mathrm{NAD}}^{+}$-MBs/GPTS/GO/NiO films immersed in 0.5 mM potassium hexacyanoferrate solution.

In this study, the different films, such as LDH-${\mathrm{NAD}}^{+}$/GPTS/NiO, LDH-${\mathrm{NAD}}^{+}$/GPTS/GO/NiO and LDH-${\mathrm{NAD}}^{+}$-MBs/GPTS/GO/NiO films, were immersed in 0.5 mM potassium hexacyanoferrate solution to investigate electrochemical impedances of modified and unmodified films. From EIS results, $R_{\mathrm{et}}$ of LDH-${\mathrm{NAD}}^{+}$-MBs/GPTS/GO/NiO film was smaller than those of LDH-${\mathrm{NAD}}^{+}$/GPTS/GO/NiO film and LDH-${\mathrm{NAD}}^{+}$/GPTS/NiO film, as shown in Figure 7. It is attributed to the electron transfer ability of LDH-${\mathrm{NAD}}^{+}$-MBs/GPTS/GO/NiO film being better than those of LDH-${\mathrm{NAD}}^{+}$/GPTS/GO/NiO film and LDH-${\mathrm{NAD}}^{+}$/GPTS/NiO film. More specifically, $R_{\mathrm{et}}$ of LDH-${\mathrm{NAD}}^{+}$/GPTS/NiO film modified by GO (i.e., LDH-${\mathrm{NAD}}^{+}$/GPTS/GO/NiO film) was about 3.2 times larger than LDH-${\mathrm{NAD}}^{+}$/GPTS/NiO film. On the other hand, $R_{\mathrm{et}}$ of LDH-${\mathrm{NAD}}^{+}$/GPTS/NiO film modified by GO and MBs (i.e., LDH-${\mathrm{NAD}}^{+}$-MBs/GPTS/GO/NiO film) was about 4.9 times larger than LDH-${\mathrm{NAD}}^{+}$/GPTS/NiO film, and it was about 1.5 times larger than LDH-${\mathrm{NAD}}^{+}$/GPTS/GO/NiO film. Therefore, it could be found that the contribution of GO was larger than MBs on electron transfer process.

### 3.5. Detection Range of Flexible Arrayed Lactate Biosensor

**Figure 8:**
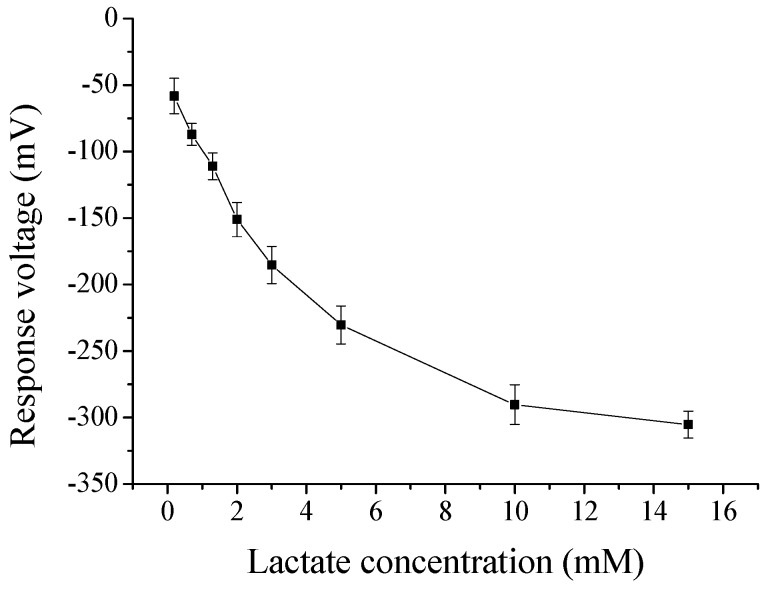
The detection of flexible arrayed lactate biosensor.

**Figure 9:**
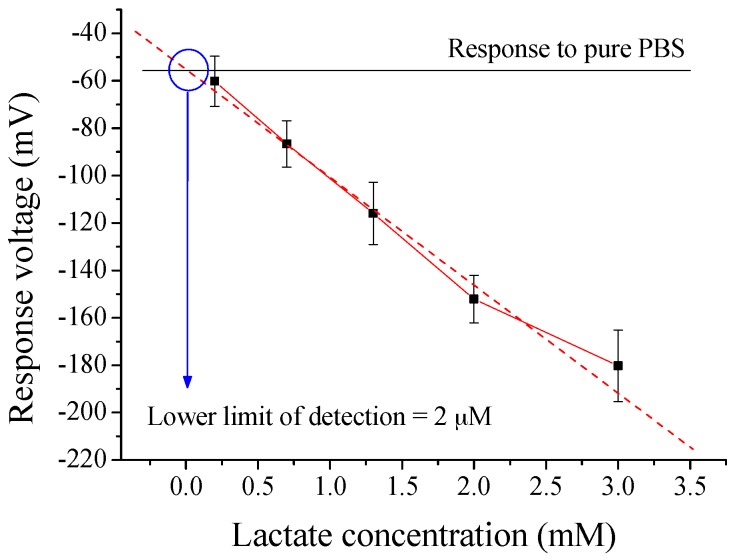
The lower limit of detection of flexible arrayed lactate biosensor. PBS: phosphate buffer solution.

According to the above, we could know that the optimal GO and MB contents were, respectively, 0.3 wt % and 0.75 mL for flexible arrayed lactate biosensor. Next, we also investigated its detection range, as shown in Figure 8. The response voltage was measured by potentiometric measurement system. We could observe that the response voltage was decreased with an increase in lactate concentration until about 15 mM. There was no significant variation on response voltage between 10 mM and 15 mM, and their error bars were inter-overlapped. After that, we could estimate its lower limit of detection by a point of intersection for two lines, where one line was the response voltage for pure PBS (not containing lactate), and another line was the trend line of experiment data. Figure 9 showed that the lower limit of detection is about 2 μM.

### 3.6. Anti-Interference Effect of Flexible Arrayed Lactate Biosensor

**Figure 10:**
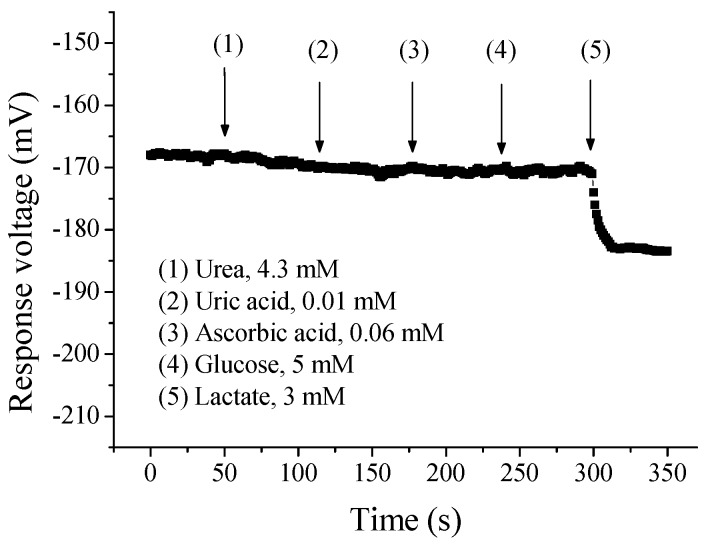
The anti-interference effect of flexible arrayed lactate biosensor against various substances.

In order to confirm the anti-interference ability of flexible arrayed lactate biosensor, the various substances were added into the test solution when monitoring. In this study, we used some substances that are commonly present in the human body, such as urea, uric acid, ascorbic acid and glucose. At the beginning of the experiment, we immersed the flexible arrayed lactate biosensor into the test solution containing the lactate, and which concentration was 1.3 mM. The response voltage was measured by potentiometric measurement system. Then, 4.3 mM urea, 0.01 mM uric acid, 0.06 mM ascorbic acid and 5 mM glucose were sequentially added into the test solution by using micropipettor because they were common substances in the human body. In addition, these concentrations belonged to the normal range in the human body. After that, 3 mM solution was added into the test solution by using micropipettor. The result of the anti-interference effect was shown in Figure 10. Even though these substances were added into 1.3 mM solution, the response voltage kept within the reasonable range. There was no obvious variation on response voltage, and which fell within the response range of 1.3 mM lactate solution. However, when 3 mM solution was added into the test solution, the response voltage was varied significantly. It exhibited excellent anti-interference ability, which would be attributed to excellent selectivity of flexible arrayed lactate biosensor.

### 3.7. Stability of Flexible Arrayed Lactate Biosensor under Bending

**Figure 11:**
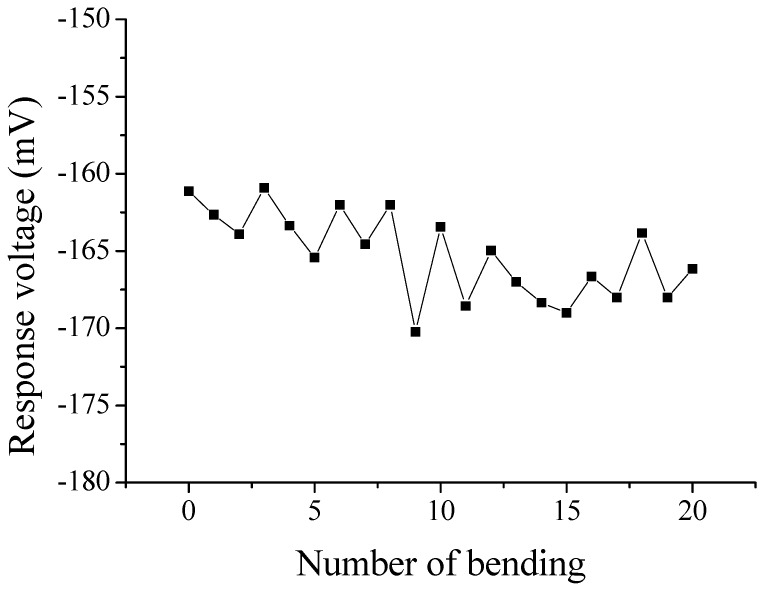
The variation on response voltage of flexible arrayed lactate biosensor after bending 20 times.

Moreover, we also investigated another important issue, i.e., the stability of flexible arrayed lactate biosensor after repeated bending. The flexible arrayed lactate biosensor was bended under U shape, as shown in Figure 2b. After that, the flexible arrayed lactate biosensor was unfolded and immersed in 1.3 mM lactate solution, and which response voltage was measured by potentiometric measurement system. The above steps were repeated 20 times. The results were shown in Figure 11. We observed the variations on response voltage of flexible arrayed lactate biosensor after each bending. After the flexible arrayed lactate biosensor was bended 20 times, there was almost no variation on response voltage. The variation of response voltage was around 10 mV, as shown in Figure 11.

### 3.8. Comparisons of Various Lactate Biosensors

Table 3 showed the comparisons of sensitivity and their concentration ranges for various lactate biosensors. In the literature [20], they immobilized the lactate oxidase (LOD) and manganese dioxide nanoparticles (${\mathrm{MnO}}_{2}$ NPs) into layer-by-layer poly(dimethyldiallylammonium chloride) (PDDA) films to fabricate (PDDA/${\mathrm{MnO}}_{2}$/PDDA/LOD)_n_ multilayer films, and which were used for the lactate biosensor based on enzyme field effect transistor (ENFET). The sensitivity was 16.84 mV/mM. In the literature [21], they proposed ${\mathrm{NAD}}^{+}$-dependent ENFET-based lactate biosensor. The aminosiloxane-functionalized gate interface was modified with pyrroloquinoline quinone (PQQ), which acted as a catalyst for the oxidation of NADH. ${\mathrm{NAD}}^{+}$ and LDH were covalently linked to the PQQ monolayer. The sensitivity was 26 mV/dec, and lower limit of detection was 0.1 mM. In the literature [22], they reported a microsensor, and which used an ENFET with a platinum microelectrode and combined potentiometric and amperometric techniques. The sensitivity could achieve 20 mV/mM. In the literature [23], they developed a lactate biosensor based on electrolyte–insulator–semiconductor using a nanostructured ${\mathrm{Si}}_{3}N_{4}$ surface modified by a polyacrylic acid (PAA) layer, and which could covalently link to the ${\mathrm{NH}}_{2}$ groups of LDH. The sensitivity was 49.7 mV/dec. Compared with the other literatures [20,21,22,23], the flexible arrayed lactate biosensor based on NiO film modified by GO and MBs exhibits excellent sensitivity though its concentration range was narrower than the other literatures [20,21,22,23].

## 4. Conclusions

The flexible arrayed lactate biosensor based on immobilizing LDH-${\mathrm{NAD}}^{+}$ on NiO film modified by GO and MBs exhibits excellent sensitivity (45.397 mV/mM) with a linearity of 0.992. Besides, it had a great anti-interference ability, and its lower limit of detection was estimated to be about 2 μM. According to EIS results, the electron transfer resistance of LDH-${\mathrm{NAD}}^{+}$-MBs/GPTS/GO/NiO film was smaller than those of LDH-${\mathrm{NAD}}^{+}$/GPTS/GO/NiO film and LDH-${\mathrm{NAD}}^{+}$/GPTS/NiO film, and it presented the outstanding electron transfer ability. Moreover, after the flexible arrayed lactate biosensor was bended 20 times, there was almost no variation on response voltage.

## Figures and Tables

**Table 1:** The average sensitivity and linearity for flexible arrayed lactate biosensors modified by different graphene oxide (GO) contents.

GO Content (wt %)	Average Sensitivity (mV/mM)	Linearity
0	38.218	0.992
0.1	39.237	0.998
0.3	40.018	0.995
0.5	37.146	0.994

**Table 2:** The average sensitivity and linearity for flexible arrayed lactate biosensors modified by different magnetic beads (MB) contents.

MB Content (mL)	Average Sensitivity (mV/mM)	Linearity
0	40.018	0.995
0.25	42.531	0.987
0.50	43.595	0.993
0.75	45.397	0.992
1.00	43.606	0.992

**Table 3:** The comparisons of sensitivity and linearity for lactate biosensors with various films.

Film	Sensitivity	Concentration Range (mM)	References
LDH-${\mathrm{NAD}}^{+}$-MBs/GPTS/GO/NiO	45.397 mV/mM	0.2–3	This study
LOD/MnO_2_ NPs/SiO_2_	16.84 mV/mM	0.1–5	[20] 2005
LDH/${\mathrm{NAD}}^{+}$/PQQ/SiO_2_	26 mV/dec	0.1–10	[21] 2001
LOD/Si_3_N_4_/SiO_2_	20 mV/mM	1–6	[22] 2013
LDH-PAA-Si_3_N_4_	49.7 mV/dec	0.2–$10^{-3}$	[23] 2007
